# Nitric Oxide Plays a Key Role in Ovariectomy-Induced Apoptosis in Anterior Pituitary: Interplay between Nitric Oxide Pathway and Estrogen

**DOI:** 10.1371/journal.pone.0162455

**Published:** 2016-09-09

**Authors:** Sonia A. Ronchetti, Leticia I. Machiavelli, Fernanda A. Quinteros, Beatriz H. Duvilanski, Jimena P. Cabilla

**Affiliations:** 1 Departamento de Química Biológica, IQUIFIB, Facultad de Farmacia y Bioquímica, Universidad de Buenos Aires, Buenos Aires, Argentina; 2 Instituto de Investigaciones Biomédicas (INBIOMED) UBA-CONICET, Facultad de Medicina, Universidad de Buenos Aires, Buenos Aires, Argentina; University of Rome, ITALY

## Abstract

Changes in the estrogenic status produce deep changes in pituitary physiology, mainly because estrogens (E2) are one of the main regulators of pituitary cell population. Also, E2 negatively regulate pituitary neuronal nitric oxide synthase (nNOS) activity and expression and may thereby modulate the production of nitric oxide (NO), an important regulator of cell death and survival. Little is known about how ovary ablation affects anterior pituitary cell remodelling and molecular mechanisms that regulate this process have not yet been elucidated. In this work we used freshly dispersed anterior pituitaries as well as cell cultures from ovariectomized female rats in order to study whether E2 deficiency induces apoptosis in the anterior pituitary cells, the role of NO in this process and effects of E2 on the NO pathway. Our results showed that cell activity gradually decreases after ovariectomy (OVX) as a consequence of cell death, which is completely prevented by a pan-caspase inhibitor. Furthermore, there is an increase of fragmented nuclei and DNA cleavage thereby presenting the first direct evidence of the existence of apoptosis in the anterior pituitary gland after OVX. NO production and soluble guanylyl cyclase (sGC) expression in anterior pituitary cells increased concomitantly to the apoptosis. Inhibition of both, NO synthase (NOS) and sGC activities prevented the drop of cell viability after OVX, showing for the first time that increased NO levels and sGC activity observed post-OVX play a key role in the induction of apoptosis. Conversely, E2 and prolactin treatments decreased nNOS expression and activity in pituitary cells from OVX rats in a time- and E2 receptor-dependent manner, thus suggesting interplay between NO and E2 pathways in anterior pituitary.

## Introduction

Maintenance of tissue homeostasis is based on the balance of three different processes: cell proliferation, differentiation and death. In these conditions, apoptosis is the main form of cell death [1].

In the anterior pituitary gland, as well as in other endocrine tissues, regulation of tissue remodelling is closely related to changes in hormonal status. In the rat, the number of anterior pituitary cells fluctuates depending on different physiological situations, especially in response to alterations in estrogen levels, given that these hormones are of significant importance in the regulation of pituitary cell populations [2–5]. Estrogens have been shown to exert proliferative actions on this gland. Enlarged pituitaries have been observed after chronic treatment with high estradiol (E2) concentrations [3,6,7] whereas cessation of this treatment induces apoptosis, the gland returning to its normal shape and size [6,8]. In relation to these reports, it has been suspected that the lack of E2 caused by ovariectomy would induce anterior pituitary cell death in the gland. However, no studies up to now have provided this evidence.

Nitric oxide (NO), a free radical gas, is a well known intracellular messenger in a wide range of physiological processes such as neurotransmission, vasodilation, and immune response [9–11]. NO also behaves as a regulator of cell death and survival. It can turn on or shut off apoptotic pathways depending largely on NO concentration and exposure time [12,13]. In fact, we previously showed that NO has a dual effect on anterior pituitary cells. It has a protective role in cells exposed to cytotoxic agents [14], but it induces apoptosis after long-term exposure of these cells to micromolar NO concentrations [15].

NO is produced by NO synthases (NOS, EC 1.14.13.39). Three isoforms of NOSs are expressed in the anterior pituitary [16,17]. Calcium-dependent neuronal NOS (nNOS) and endothelial NOS (eNOS) are constitutively expressed, although their activity and expression can be regulated by different stimuli. nNOS is the most abundant isoform localized in gonadotrophs and follicle stellate cells [16] whereas eNOS is confined to endothelial cells. The calcium-independent, inducible isoform (iNOS) is not detected in normal conditions in anterior pituitary cells [18].

NOSs are regulated by E2 [19–21]. This hormone can act on pituitary cells in a direct or indirect fashion by modifying neurotransmitter release and hypothalamic peptides or intra-pituitary factors. E2 directly exerts a stimulatory effect on eNOS [22], whereas it down-regulates nNOS through inhibition of gonadotropin-releasing hormone (GnRH) at hypothalamic level [23]. However, the possibility of E2 direct regulation of pituitary nNOS has not yet been addressed.

Several studies in rats have shown that after E2 withdrawal, mRNA and protein levels of pituitary nNOS increase, as do the number and size of gonadotrophs (nNOS-expressing cells) and NOS activity [16,23–25]. Consistently, substitution treatment with E2 completely prevents these increases [16,25], and chronic E2 treatment down-regulates nNOS expression [26]. Additionally, cGMP levels were shown to be augmented in 14 days-OVX rats [25]. Altogether, these findings suggest a negative E2 regulation on NO pathway.

NO exerts most of its physiological effects by stimulating soluble guanylyl cyclase (sGC, EC 4.6.1.2). This cytosolic NO receptor catalyses the conversion of GTP to cyclic GMP (cGMP), a very important intracellular second messenger [27,28].

sGC is an heterodimeric enzyme and is comprised of two subunits, α and β, of which four types exist (α1, α2, β1, and β2). The α1/β1 is the most abundant and widely expressed heterodimer, and shows greatest activity [29,30]. Many α1 and α2 splicing variants have been described [31]; among them α2 and α2i are expressed in anterior pituitary cells and play an important role in regulation of sGC activity [30]. We previously showed that E2 directly down-regulates NO pathway in anterior pituitary by modifying sGC expression and by decreasing its activity [29,30]. Overall, these findings together with evidence of NOS indirect regulation by E2 underline that this hormone regulates NOS/NO/sGC/cGMP pathway at multiple levels in anterior pituitary gland.

E2, as a major regulator of anterior pituitary physiology, up-regulates prolactin (PRL) synthesis and release [32]. Also, previous reports from our lab demonstrated that NO inhibits PRL release [25,33] thus suggesting interplay between E2 and NO pathways.

In order to elucidate the complex map of interactions between NO and E2 pathways in anterior pituitary gland, we aimed to investigate:

1. the role of NOS/NO/sGC/cGMP pathway in anterior pituitary cell death induced after OVX *in vivo*;
2. whether E2 or a downstream effector (PRL) directly affects NO pathway by acting on nNOS expression and activity *in vitro*.

## Materials and Methods

### Ethics Statement

All experimental procedures were approved by the Institutional Animal Care and Use Committee (IACUC) of the School of Medicine (University of Buenos Aires, Resolution No. 2831/10) and were carried out in compliance with the guidelines of the NIH Guide for the Care and Use of Laboratory Animals.

### Drugs and reagents

Ketamine and xylazine were purchased from Holliday-Scott and König, respectively (Buenos Aires, Argentina). Ketofen was obtained from Vetanco S.A. (Buenos Aires, Argentina). Media and reagents for cell culture were purchased from Gibco (Rockville, MD, USA), except for the fetal bovine serum which was obtained from GBO (Buenos Aires, Argentina). Go Taq DNA polymerase, random hexamers and dNTPs were provided by Promega (Madison, WI, USA). TRIzol and molecular biology reagents were from Invitrogen (Carlsbad, CA, USA). Unless stated otherwise, all other reagents and antibodies were obtained from Sigma (St. Louis, MO).

### Animals

Adult female Wistar rats weighing 200–250 g (Bioterio Central, Facultad de Farmacia y Bioquímica, Universidad de Buenos Aires) were kept on a 12 h light-dark cycle with controlled temperature (20–22°C) in accordance with the NIH Guide for the Care and Use of Laboratory Animals. Food and water were supplied ad libitum. The rats were ovariectomized under 75 mg/kg ketamine and 10 mg/kg xylazine anesthesia 4, 7 and 14 days before the experiments. After surgery and the following day, animals received a s.c. injection of 1% ketoprofen. Sham operated rats were sacrificed by decapitation at random stages of estrous cycle were used as control (N = 6 rats per group).

### Cell culture

Primary cell cultures -constituted of all secretory and non-secretory pituitary cell types- were prepared from anterior pituitary glands from control and ovariectomized (OVX) rats killed at different times post-surgery. Anterior pituitary glands (6 rats per group) were removed within minutes after decapitation and pooled for each cell culture. Cells were obtained by enzymatic (trypsin/DNase) and mechanical dispersion (extrusion through a Pasteur pipette). In all experimental conditions, cell viability was assessed by trypan blue exclusion and was always >90%. In all cases, dispersed cells were seeded onto tissue culture plates and stabilized for 24 h (37°C, 5% CO_2_ in air) in phenol red-free Dulbecco’s modified Eagle’s medium (DMEM) supplemented with 10% charcoal stripped fetal bovine serum (CSFBS), 10 μL/mL MEM amino acids, 2 mM glutamine, 5.6 μg/mL amphotericin B, and 25 μg/mL gentamicin (DMEM-S-10% CSFBS).

### Cell treatment

After the stabilization period (24 h), medium was replaced with fresh medium and cells were incubated for 48 h (37°C, 5% CO_2_ in air) with different drugs. After treatments, the corresponding determinations were carried out. Control of treatments was performed by incubating cells in medium without drugs.

### Cell activity assay

For cell activity experiments, 0.1 × 10^6^ cells/well from each experimental condition were seeded onto 96-well tissue culture plates. After the different treatments, cell activity was assessed by methylthiazolyldiphenyl-tetrazolium bromide (MTT) assay as previously described [34]. Optical density was determined at 600 nm by an ELISA plate reader (Biotrak II, Amersham Biosciences, USA). Cell activity was measured as an index of cell viability and expressed as percentage of control.

### Microscopic analysis of nuclear morphology

For this study, 0.1 × 10^6^ cells/well were seeded on glass coverslips onto 24-well culture plates, fixed in 4% formaldehyde for 30 min at 4°C and mounted in anti-fade solution containing 1 μg/mL 4,6-diamidino-2-phenylindole (DAPI), 23.3 mg/mL 1,4-diazabicyclooctane (DABCO), and 20 mM Tris HCl (pH 8) in glycerol. Nuclear morphology was visualized and quantified in a fluorescence microscope (Olympus BX50, Japan). At least 500 nuclei were counted from random fields of each coverslip. Three coverslips per experimental group were analyzed. Results were expressed as (number of apoptotic nuclei/total number of nuclei) × 100.

### Analysis of DNA content

A freshly suspension of 1 × 10^6^ cells/mL was prepared in calcium- and magnesium-free Krebs buffer. One mL of each suspension was transferred to a 6-ml polypropylene tube (per triplicate). Cells were centrifuged at 900 × g for 10 min, fixed and permeabilized with 70% ice-cold ethanol at 4°C for 2 h. Cells were centrifuged at 900 × g for 10 min and pellets resuspended in freshly prepared propidium iodide (PI) staining solution (10 μg/mL RNase A and 50 μg/mL PI in PBS) and incubated at 37°C for 15 min, and at 4°C overnight. PI fluorescence analysis was done in a Becton Dickinson FACScalibur flow cytometer (San Jose, CA, USA), ex λ: 488 nm, em λ: 600 nm, FL2; 1 × 10^5^ cells were measured per tube. Data were analyzed using WinMDI 2.8 software.

### Nitrite determination

Cells (0.5 × 10^6^/0.5 mL/well) were seeded onto 24-well culture plates. Nitrite concentration in the culture media was measured by the nitrate reductase-Griess assay [35]. A standard curve with sodium nitrite was performed. As culture media contain basal levels of nitrites and nitrates, parallel reactions were performed with culture medium alone and absorbance value obtained was subtracted from all sample values. Results were calculated as concentration of nitrites (μM) in the incubation medium.

### Protein determination

Protein content was assayed by the Bradford method (BioRad, Buenos Aires, Argentina) using bovine serum albumin as standard.

### Immunoblot analysis

Fifty micrograms of total protein from each sample was boiled for 5 min in Laemmli sample buffer and size-fractioned on 10% SDS-PAGE. Resolved proteins were transferred to polyvinylidene difluoride membranes and blocked for 1–2 days at 4°C in blocking buffer (TBS-0.05% Tween 20, 6% nonfat dry milk). Membranes were co-incubated overnight at 4°C with rabbit antisera anti-sGC α1 (G4280, 1:1750), anti-sGC β1 (G4405, 1:700), anti-nNOS (610310, 1:200) or anti-caspase 3, active (C8487, 1:200) together with anti- β-actin (A2066, 1:1000) in blocking buffer. Blots were washed and incubated for 1 h at room temperature with horseradish-peroxidase conjugated goat antirabbit IgG (1:2000), followed by detection of immunoreactivity with diaminobenzidine solution containing 0.01% hydrogen peroxide. Bands were quantified with Gel Pro Analyzer (Media Cybernetics, LP, Silver Spring, MD, USA) software for Windows. Intensity data were normalized with respect to the corresponding β-actin blot in each lane.

### RNA isolation

177 μL of TRIzol reagent was added to each well. After isolation, total RNA from tissues was spectrophotometrically quantified at 260 nm. RNA integrity was checked in formaldehyde/formamide gel electrophoresis.

### RT and PCR reactions

First strand cDNA was synthesized with Moloney murine leukemia virus (M-MLV) reverse transcriptase in RT buffer containing 5.5 mM MgCl_2_, 0.5 mM dNTP, 2.5 μM random hexamers, and 3.125 U/μL M-MLV reverse transcriptase. Reactions were done in a final volume of 12 μL containing 1 μg RNA. The reverse transcription reaction was run at 37°C for 50 min and reverse transcriptase was inactivated by heating the samples at 70°C for 15 min before the PCR reactions. To check for genomic contamination, the same procedure was performed on samples in a reaction solution lacking reverse transcriptase.

Specific primers were designed from published sequences [30] and are detailed in Table 1.

**Table 1:** Primers used for semi-quantitative RT-PCR assays.

Gene	Locus	Primers	Product size (bp)
sGCα2	NC_005107.2	5’-GCGACTGTCTACCCCGTTTGTGAT-3’	399
		5’-CTGTACTTGCTGCCCTTGCCATAA-3’	
sGCα2i	NM_017090	5’-TTTTCTCCTTTCCTGTTTCCATCC-3’	275
		5’-ACGAGACCGCGGAATGAATG-3’	
nNOS	NM_052799.1	5’-ATCGGCGTCCGTGACTACTG-3’	867
		5’-TCCTCATGTCCAAATCCATCTTCTTG-3’	
β-actin	NM_031144	5’-ACCACAGCTGAGAGGGAAATCG-3’	276
		5’-AGAGGTCTTTACGGATGTCAACG-3’	

Amplified products spanned from nucleotide position bases 1530 to 1929 in the C- terminal of sGC α2 (NC_005107.2), from the in frame insert to 275 bp to 3’ end of sGC α2_i_ (NM_017090) and from 1878 to 2745 of nNOS (NM_052799.1). β-actin (NM_031144) was used as an endogenous control. Actin primers were designed in order to detect amplification of DNA contamination. Then, samples were thermocycled for PCR amplification (Mastercycler, Eppendorf, Hamburg, Germany). The reaction mixture contained GoTaq PCR buffer, 1.5 mM MgCl_2_, 200 μM of each dNTP, 0.625 U GoTaq polymerase and 300 nM of each primer. We utilized RT-PCR methods to determine relative changes in mRNA expression. Reactions were subjected to a varying number (n = 16–40) of cycles of PCR amplification (melting phase 94°C for 30 sec, annealing 55°C for 30 sec and extension 72°C for 1 min) to determine the optimum cycle number within the linear range for PCR amplification. Amplified products collected from various cycles were analyzed by electrophoresis in 1.5% agarose-ethidium bromide gels, and the optimum cycle number was found to be 24 cycles for β-actin, and 40 cycles for sGC α2, sGC α2_i_ and nNOS. The intensity of PCR products was determined by digital image analysis using Gel Pro Analyzer (Media Cybernetics, LP, Silver Spring, MD) software for Windows. sGC subunit levels were normalized to the value of the β-actin amplified band in each lane.

### Statistical analysis

Results were expressed as mean ± SE and evaluated by one-way ANOVA followed by Tukey’s or Dunnett’s tests depending on the experimental design. Differences between groups were considered significant if p<0.05. Results were confirmed by at least three independent experiments.

## Results

### E2 depletion induces apoptosis in anterior pituitary cells

We initiated our study by assessing to what extent the removal of basal E2 stimulus affects anterior pituitary cells. Therefore, we carried out an MTT assay in order to determine cell viability in primary cultures of anterior pituitary cells from OVX rats sacrificed 4, 7 and 14 days after surgery.

**Fig 1:**
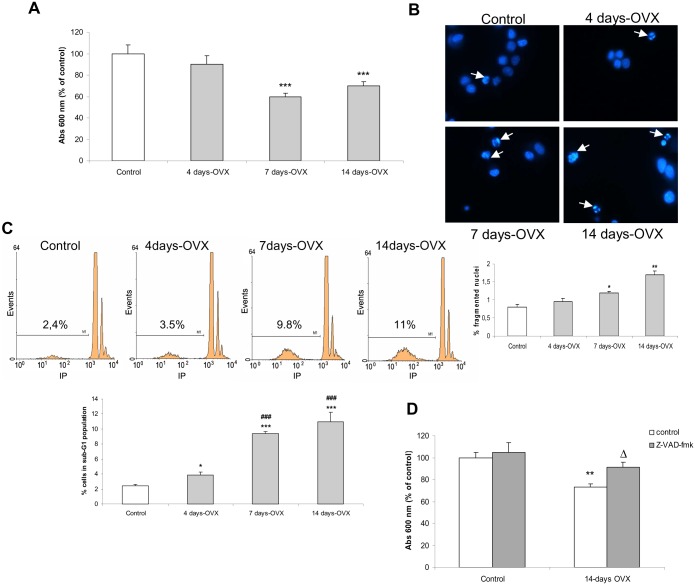
Ovariectomy reduced anterior pituitary cell viability. Anterior pituitary primary cell cultures from control and 4, 7 and 14 days-OVX rats were stabilized for 24 h and incubated for another 48 h (**A**, **B** and **D**) or freshly dispersed after killing (**C**). (**A**), cell viability was assessed by MTT assay. (**B**), nuclear morphology was studied by DAPI staining. (**C**), DNA content was analyzed by flow cytometry. (**D**), control and 14 days-OVX pituitary cells were incubated with or without 50 μM z-VAD-fmk (a pan-caspase inhibitor) and cell viability was determined by MTT assay. Results represent mean absorbance (**A,D**) or fluorescence (**C**) ± SE (N = 3), and were expressed as percentage of control. ANOVA followed by Tukey’s test, *p<0.05, **p<0.01, ***p<0.001 vs. control; ###p<0.001 vs 4 days-OVX; Δp<0.05 vs. 14 days-OVX.

Cell viability significantly decreased after 7 days-OVX and this reduction was unchanged in cultures until 14 days-OVX (Fig 1A). These results showed that depletion of basal E2 stimulus negatively affects viability of anterior pituitary cells.

In order to study whether reduction in anterior pituitary cell viability might be due to apoptosis, we first examined nuclear morphology from OVX rats sacrificed at different times post surgery. A significant increase in the percentage of fragmented nuclei was observed in 7 days-OVX rats, and was markedly augmented after 14 days-OVX (Fig 1B).

Apoptotic cells undergoing DNA cleavage are susceptible to extensive loss of DNA content, and consequently a distinct population of cells with hypodiploid DNA content (sub-G1 population) can be observed by flow cytometry analysis. Control and 4, 7 and 14 days-OVX rats were sacrificed and their pituitaries processed for analysis of DNA content. After 4 days-OVX, a slight increase in the percentage of cells in the sub-G1 population was observed, which became significantly higher after 7 and 14 days (Fig 1C).

Caspase activation is a common point of several pro-apoptotic stimuli. To address whether caspases were involved in the apoptosis induced in anterior pituitary after E2 removal, cell cultures from control and 14 days-OVX rats were incubated for 48 h with or without Z-VAD-fmk, a pan-caspase inhibitor, then cell viability was determined by MTT assay. The caspase inhibitor did not affect the viability of cells from control rats. However, it impeded reduction in viability of cells from OVX rats (Fig 1D), suggesting that the apoptotic process induced by E2 depletion is dependent on caspase activation.

All together, these results strongly suggest that reduction in cell viability produced after E2 removal is a consequence of induction of apoptosis in anterior pituitary cells.

### NO production increases after E2 removal

It has been demonstrated that nNOS expression and activity increase in anterior pituitary gland after OVX [23,25]. However, pituitary NO production after E2 depletion has not been studied. NO levels were quantified by the accumulation of nitrates and nitrites (NOx) in incubation media of anterior pituitary cell cultures from OVX rats sacrificed 4, 7, and 14 days after OVX.

**Fig 2:**
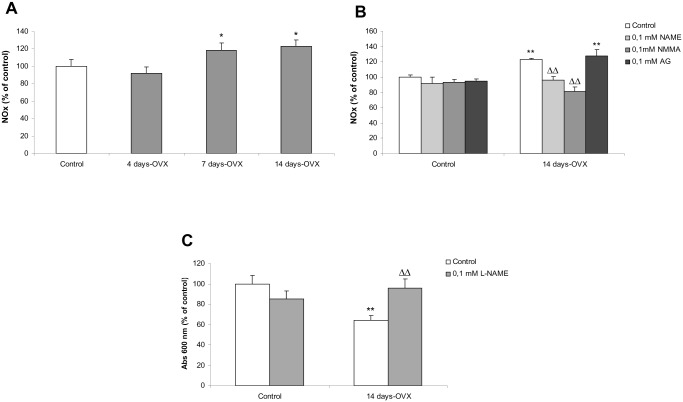
nNOS-derived pituitary NO production plays an important role in OVX-induced apoptosis. **(A)** Anterior pituitary primary cell cultures from control and 4, 7 and 14 days-OVX rats were stabilized for 24 h. (**B**) control and 14 days-OVX pituitary cells were incubated with or without 0.1 mM L-NMMA (a non-selective NOS inhibitor), 0.1 mM L-NAME (a more selective nNOS inhibitor) or 0.1 mM AG (an iNOS selective inhibitor). NOx concentration was determined in the culture media by nitrate reductase-Griess assay after 48 h. (**C**) control and 14 days-OVX pituitary cells were incubated with or without 0.1 mM L-NAME. Cell viability was determined by MTT assay. Results represent mean ± SE (N = 3) and were expressed as percentage of control. ANOVA followed by Tukey’s test, *p<0.05, **p<0.01 vs. respective control; ΔΔp<0.01 vs. 14 days-OVX rats.

A significant increase in NOx concentration was observed after 7 days-OVX, which remained elevated until 14 days (Fig 2A).

### nNOS stimulation mediates the increase in NO levels

In order to identify the specific NOS isoform responsible for NO increase after OVX, NOx concentration was quantified in incubation media of anterior pituitary cell cultures from control and 14 days-OVX rats after treatment with different NOS inhibitors.

As expected, NMMA (a non-selective NOS inhibitor) treatment prevented the rise in NOx observed after 14 days-OVX (Fig 2B). The same results were obtained when cells were incubated with L-NAME (an inhibitor with major selectivity for nNOS). However, treatment with aminoguanidine (AG), a selective iNOS inhibitor, did not modify the levels of NOx achieved after 14 days-OVX (Fig 2B).

Together these results indicate that the increase in NOx levels produced after 14 days after E2 removal would be mainly mediated by pituitary nNOS. The iNOS isoform seemed not to be involved in NO production, but we could not rule out the contribution of eNOS, since the nNOS inhibitor used (L-NAME) reacted in some extent with the eNOS isoform.

### Pituitary NO plays an important role in the OVX-induced apoptosis

As NO levels increased simultaneously with induction of apoptosis after the loss of E2 stimulus, and considering that NO is able to induce apoptosis in the anterior pituitary cells [15], it is possible that NO may be involved in the execution of cell death during the E2 depletion period. To further assess this hypothesis, anterior pituitary cell cultures from control and 14 days-OVX rats were incubated with or without L-NAME for 48 h and then cell viability was determined by MTT assay. L-NAME treatment prevented the decrease in viability of cells from 14 days-OVX rats whereas it did not affect viability of cells from control rats (Fig 2C).

These results strongly suggest that the higher levels of NO achieved in the anterior pituitary after OVX play an important role in the induction of apoptosis in anterior pituitary cells.

### sGC mediates NO-induced pituitary cell death

**Fig 3:**
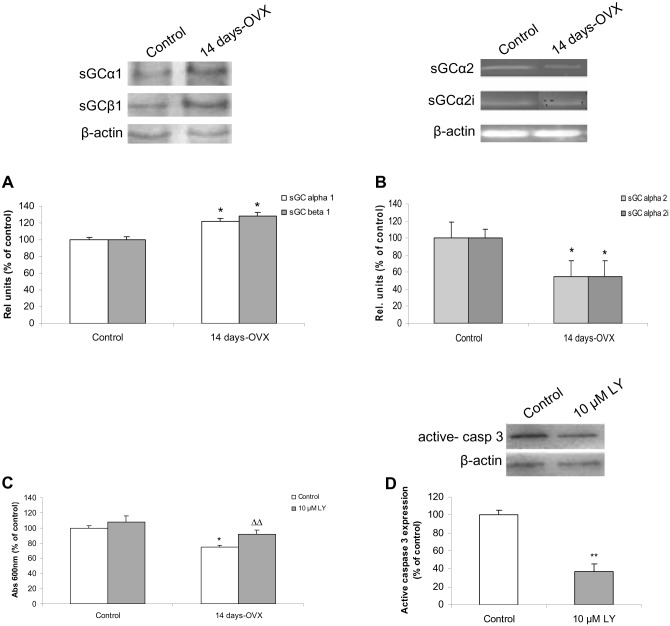
sGC expression is augmented after OVX and mediates NO-induced pituitary cell death. Anterior pituitary primary cell cultures from control and 14-days OVX rats were stabilized for 24 h. **(A)** α1 and β1 protein levels were measured by western blot after 48 h. **(B)**, α2 and α2i mRNA levels were determined by RT-PCR. (**C, D**) Cells were incubated with or without 10 μM LY-83583 (a sGC inhibitor) for 48 h. (**C**) Cell viability was determined by MTT assay. (**D**) Active caspase-3 protein levels were measured by western blot. Results represent mean ± SE, (N = 3) of relative units corresponding to the protein or mRNA densitometric values relative to β-actin expressed as percentage of control. ANOVA followed by Tukey’s test, *p<0.05, **p<0.01 vs. control, ΔΔp<0.01 vs. 14 days-OVX rats.

NO exerts most of its physiological effects through the activation of its intracellular receptor, soluble or nitric oxide-sensitive guanylyl cyclase (sGC). Previous reports indicate that after ovariectomy, sGC activity is augmented [25], consequently we studied how E2 depletion affects sGC expression. Protein levels of α1 and β1 sGC subunits were determined in anterior pituitaries from control and 14 days-OVX rats. Both α1 and β1 sGC expression significantly increased compared to control whereas the α1:β1 ratio remained similar to controls (Fig 3A). We also investigated the expression of other α isoforms such as α2 and α2i, which have been described in anterior pituitary and negatively regulate sGC activity [30]. The mRNA expression of both α2 and α2i significantly decreased after 14 days-OVX (Fig 3B), which correlates with higher activity of sGC under this condition. Together, these results indicate that the remotion of the negative E2 influence on NO pathway enhances sGC expression and activity in anterior pituitary.

Next, to ascertain whether activation of sGC is involved in OVX-induced apoptosis, anterior pituitary cell cultures from control and 14 days-OVX rats were incubated for 48 h with or without LY-83583, a sGC inhibitor, and cell viability was determined by MTT assay. The inhibitor did not affect *per se* cell viability from control rats. However, it was able to prevent reduction in viability of cells from 14-days OVX rats (Fig 3C). To further confirm these results, active caspase-3 protein levels were measured. LY-83583 treatment was able to significantly decrease its levels in 14 days-OVX rats (Fig 3D). As expected, active caspase-3 expression was almost undetectable in control rats (data not shown). These results indicate that the apoptotic cell death observed after OVX may be exerted via the NO/sGC/cGMP pathway.

### E2 inhibits nNOS expression and NO production in anterior pituitary cells

**Fig 4:**
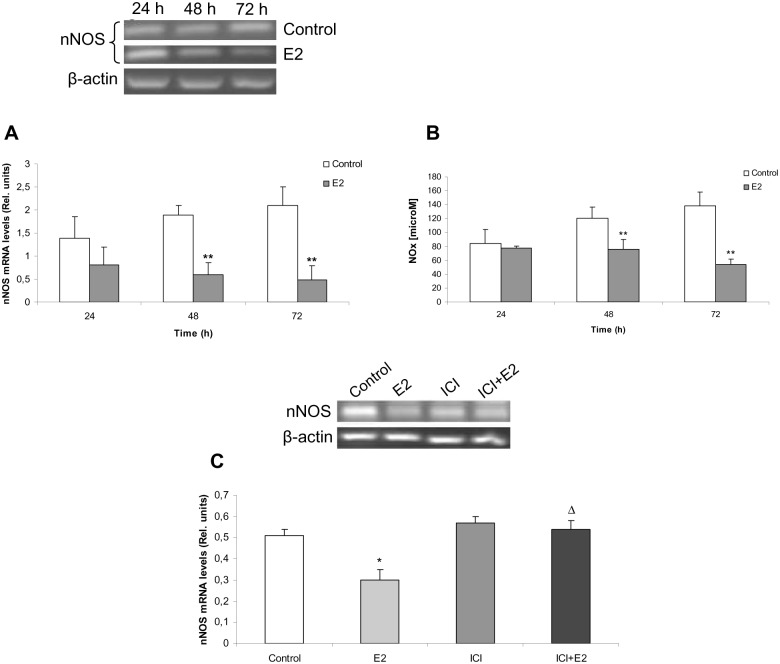
E2 down-regulates nNOS expression and activity in anterior pituitary cells through ER. Anterior pituitary cells from 14 days-OVX rats were stabilized for 24 h and incubated with or without 1 nM E2 for 24, 48 or 72 h. (**A**) nNOS mRNA levels were determined by RT-PCR. (**B**), NOx concentration was assayed by nitrate reductase-Griess assay. (**C**), Pituitary cell cultures from OVX rats were incubated with 1 nM E2 and/or with 100 nM ICI 182,780 (an estrogen receptor antagonist), 30 min before E2 treatment. nNOS mRNA levels were determined by RT-PCR. Results represent media ± SE of densitometric values relative to β-actin or nitrite concentration (N = 3). ANOVA followed by Tukey’s test, *p<0.05, **p<0.01 vs. respective control, Δp<0.05 vs. E2.

Up to this point, we have shown that E2 withdrawal led to apoptosis through NO pathway activation by increasing nNOS activity, NO levels and sGC expression and activity. E2 was previously reported to indirectly exert inhibitory effects on nNOS through GnRH [23]. However, so far there is no evidence on direct interplay between E2 and NO pathway in anterior pituitary. We investigated E2 actions on nNOS mRNA expression and activity in this gland. Anterior pituitary cells cultures from 14 days-OVX were incubated for 24, 48 and 72 h with or without E2. nNOS expression significantly diminished after 48 h and 72 h of E2 incubation (Fig 4A), which tallies with a decreased NOx production at the same time points (Fig 4B). This effect was E2-specific and dependent on E2 receptor (ER), since it was completely impeded by treatment with ICI 182,780, an ER competitive antagonist (Fig 4C). These results show for the first time that E2 directly reduces nNOS mRNA expression and NO production in anterior pituitary cells, no extra-pituitary factors being required to elicit this effect.

### PRL mediates E2-induced decrease of nNOS expression and NO production in anterior pituitary cells

**Fig 5:**
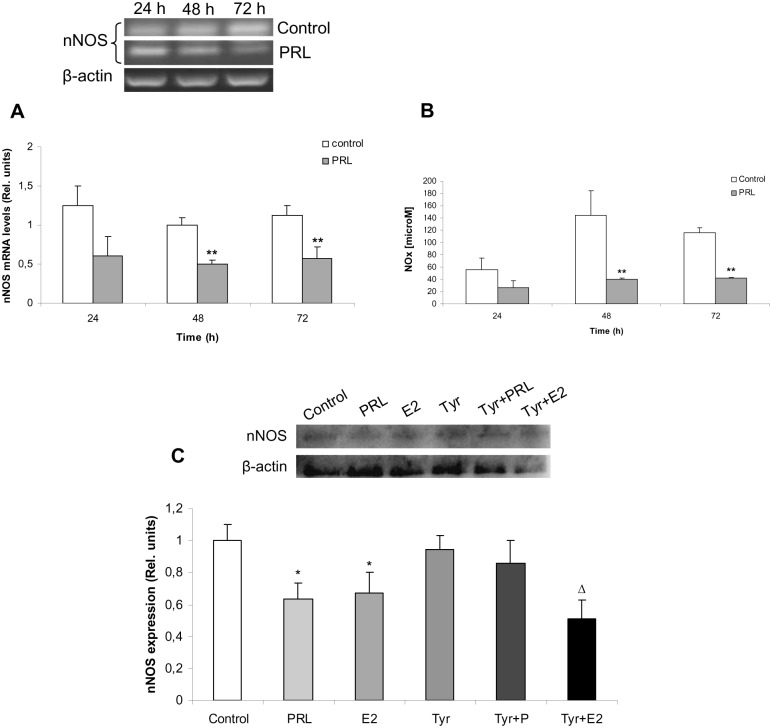
Prolactin (PRL) down-regulates nNOS expression and NO production in anterior pituitary cells. **(A, B)** Anterior pituitary cells from 14 days-OVX rats were stabilized for 24 h and incubated with or without 500 ng/mL PRL for 24, 48 or 72 h. (**A**) nNOS mRNA levels were determined by RT-PCR. **(B)**, NOx concentration was assayed by nitrate reductase-Griess assay. (**C**) Pituitary cell cultures from 14 days-OVX rats were incubated for 48 h with 20 μM Tyrphostin AG490 (Tyr), a Jak2 protein kinase inhibitor, 30 min before 1 nM E2 or 500 ng/mL PRL treatments. nNOS protein levels were determined by western blot (N = 3). Results represent media ± SE of densitometric values relative to β-actin or nitrite concentration (N = 3). ANOVA followed by Tukey’s test, *p<0.05, **p<0.01 vs. respective control; Δp<0.05 vs. Tyr.

Prolactin (PRL) is a pituitary hormone involved in several physiological functions, including reproduction, homeostasis and lactogenesis. PRL synthesis, storage and release are stimulated mainly by E2 [32,36]. Therefore, we addressed whether PRL could mediate inhibitory effects of E2 on nNOS expression and NO production. Anterior pituitary cell cultures from 14 days-OVX rats were incubated with or without PRL for 24, 48 or 72 h and nNOS expression and NOx production were assessed. Both nNOS mRNA (Fig 5A) and NOx levels (Fig 5B) decreased time-dependently after 48 and 72 h of PRL incubation.

To examine whether this effect is PRL-specific, cells were incubated with 20 μM Tyrphostin AG490 (T3434, Tyr), a Jak-2 protein tyrosine kinase inhibitor. Jak-2 is associated to both PRL receptor isoforms (L and S) and is the first step in PRL signalling. Tyr did not modify *per se* nNOS protein levels but was able to fully block PRL-induced nNOS decrease, indicating a PRL-specific effect (Fig 5C). Unexpectedly, Tyr did not impede E2 inhibitory effect on nNOS expression when co-incubated with this hormone.

These results indicate that PRL has the same effects as E2 on NO pathway, suggesting that PRL could be a mediator of E2 actions in anterior pituitary. Furthermore, E2 inhibitory actions on nNOS could take place by other PRL-independent mechanisms.

## Discussion

E2 is the major sex hormone involved in pituitary cell population regulation. Changes in the E2 status are of significant importance since it may trigger either proliferation or cell death in several E2-responsive tissues [37] such as in anterior pituitary gland [38,39]. The proliferative actions of E2 on anterior pituitary cells and specially on lactotrophs have been widely reported, whereas E2 removal decreases lactotroph population [5]. A clinical study in female patients has shown a negative correlation between pituitary volume and age and has been primarily attributed to decreasing levels of gonadal steroids after puberty [40,41]. With this evidence in mind, we hypothesized that E2 depletion after OVX negatively affects anterior pituitary cell population.

All these effects were observed after the 7^th^ day but not at the 4^th^ day of removal of ovary hormones. It is likely that at this time point E2-induced cell survival signalling remains active.

E2 exerts tissue-specific regulatory action on NOSs [19–21]. For example, E2 has been shown to stimulate nNOS expression and activity in primary neurons of the rat preoptic region [42]. However, previous reports have demonstrated an increase in nNOS expression and activity after gonadectomy [24,25,43] which are reverted by E2 administration *in vivo*, suggesting that this hormone is the main factor responsible for pituitary NOS inhibition [16]. In concordance with this evidence, here we showed that NO production increased in anterior pituitary after 7 days-OVX and remained at those levels until 14 days-OVX. In this sense, it is likely that after ovariectomy, E2-driven nNOS inhibition is suppressed, thereby stimulating enzymatic activity and consequently, NO production.

Our results demonstrated that NO production after OVX is due mainly to nNOS activity. Treatment of cells with NMMA (a NOS non-selective inhibitor) or with L-NAME (a NOS inhibitor with higher specificity for nNOS) prevented the rise in nitrite levels observed after 14 days-OVX. Because L-NAME reacts to some extent with eNOS, we cannot completely rule out that this enzyme may also be responsible for the increase in NO. However, western blot analysis of anterior pituitary samples from OVX rats showed no expression of eNOS protein in this situation [23]. Moreover, it has been shown that acute E2 administration to OVX rats increases eNOS mRNA in the gland [22], indicating that E2 stimulates eNOS expression as observed in other tissues [44,45]. With regard to iNOS, treatment with aminoguanidine (a selective iNOS inhibitor) did not modify NO production reached after 14 days-OVX, indicating that this isoform is not involved in OVX-induced NO increase. Supporting our data, studies of OVX rats showed that iNOS mRNA and protein are not detected after OVX [22,23] suggesting that this enzyme is not expressed in this particular condition. Taken together, our results indicate that the stimulation of nNOS after the abolition of its estrogenic inhibition would be the main factor responsible for the rise in NO production in anterior pituitary cells.

The direct relationship between the rise of NO levels and the decrease in cellular viability after OVX is noteworthy. Inhibition of NO synthesis prevented the decrease in the viability of cells from 14 days-OVX rats, showing for the first time that the increase of NO levels plays a central role in anterior pituitary cell death. Apoptotic effects of NO have been associated mainly with high and/or sustained concentrations produced by iNOS, whereas antiapoptotic actions linked to low NO concentrations derived from nNOS and eNOS activities [46]. In contrast with this evidence, our findings indicate that nNOS-derived NO production triggers apoptosis. In this scenario, it seems possible that OVX-induced nNOS stimulation produces low but persistent NO levels that can locally reach high concentrations, responsible for inducing apoptosis in the gland. However, since it is also possible that pituitary cells may display more sensitivity to certain stimuli as a consequence of gonadal steroid removal, lower NO levels produced by nNOS could be enough to trigger cell death.

Involvement of the sGC in the apoptotic effect of NO is controversial. While some studies show that activation of sGC mediates cytoprotective effects of NO [47,48], other reports present evidence that the activation of the sGC/cGMP pathway mediates NO apoptotic effects [49,50]. Our results showed that inhibition of sGC completely prevented the reduction in viability of cells from 14-days OVX rats, suggesting that sGC activation mediates the NO apoptotic effect in anterior pituitary. This finding, together with previous results from our laboratory showing that NO exposure increases cGMP production in anterior pituitaries of 14 days-OVX rats [25], strongly suggest that the apoptosis induced by removal of ovarian hormones could be exerted via the NO/sGC/cGMP pathway. Studies are currently underway to elucidate downstream mechanisms activated by the wide range second messenger cGMP. In this direction, cGMP-induced protein kinase G activation has been linked to NO-induced apoptosis in different tissues [49,51]. We previously demonstrated that NO, at micromolar concentrations, induces anterior pituitary cells apoptosis, and sGC was not involved in this effect [15] probably because NO, at high concentrations, inactivate this enzyme [52]. Here, all on the contrary, sGC pathway participates in NO apoptotic effect, suggesting that concentrations of NO observed were lower (than micromolar) but enough to stimulate sGC apoptotic pathway.

The opposing roles of E2 and NO pathway in anterior pituitary gland are well known although to date their interactions were not fully elucidated. It has been demonstrated that E2 through inhibition of GnRH release was shown to down-regulate NO pathway in pituitary gland by decreasing nNOS expression [23]. In addition, E2 directly inhibits sGC activity by differentially modifying sGC subunit expression [29,30]. Also, NO pathway through cGMP inhibits PRL release [33], whose synthesis and release is in turn stimulated by E2.

To complete the landscape of interactions between E2 and NO pathway in anterior pituitary gland, we addressed the *in vitro* effect of E2 on nNOS expression and activity. In this study, we demonstrated that E2 directly decreases both, nNOS expression and activity in anterior pituitary cells, thereby providing the first evidence of a direct E2-driven nNOS downregulation, unmediated by any extrapituitary factor. This effect was shown to be mediated by estrogen receptor (ER), since it was totally blocked by the specific ER antagonist ICI 182,780. Surprisingly, PRL was shown to elicit the same E2 inhibitory actions on pituitary nNOS expression and activity, which adds a novel direct regulation point between E2 and NO pathways in anterior pituitary gland. The effect of PRL on different NOS isoforms has been only scantily studied in other tissues [53–55]. To our knowledge, this is the first study showing a direct PRL-driven nNOS down-regulation. Moreover, considering that E2 inhibits nNOS expression even in the presence of a PRL receptor inhibitor, it is very likely that nNOS inhibition takes place through other PRL-independent E2 signalling. Further studies are required to elucidate mechanisms involved in this effect.

**Fig 6:**
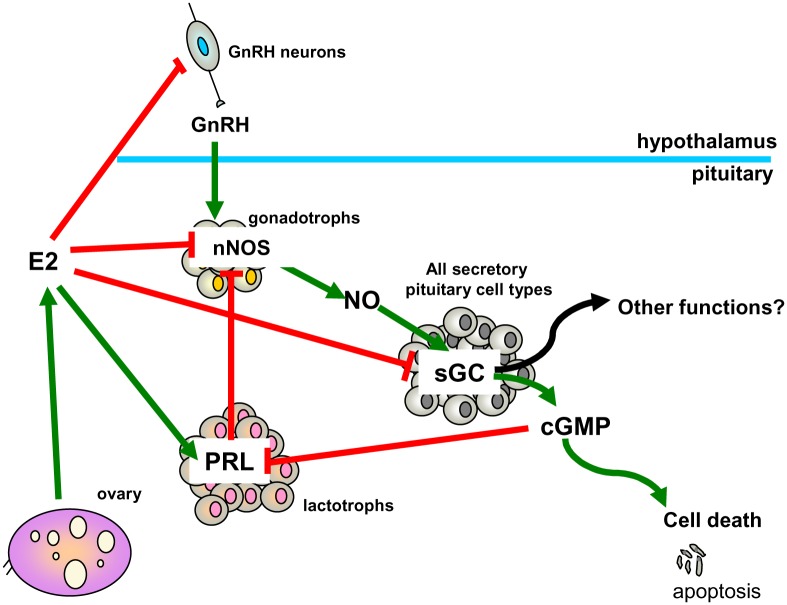
E2 and NO pathway cross-talk in anterior pituitary cells. In anterior pituitary cells, nNOS-mediated NO production is primarily associated with cell death by apoptosis. On the other hand, E2 is the main stimulator of PRL secretion, and promotes lactotroph survival and proliferation. NO and E2 pathways reciprocally inhibit each other at multiple points. NO through cGMP inhibits PRL secretion. E2 inhibits nNOS through hypothalamic GnRH inhibition and further down-regulates nNOS either directly or indirectly at pituitary level, through PRL stimulation. In addition, E2 inhibits sGC activity and promotes an imbalance in its subunits’ expression which has been associated with other functions, not related to cGMP production.

The convergence of direct and indirect regulation at multiple points of E2 and NO pathways strongly underscores their importance in pituitary cell population control. A schematic model of pituitary E2 and NO pathway cross-talk is depicted in Fig 6.

Elucidation of mechanisms that regulate anterior pituitary cell death during gonadal hormone deficiency will allow, to some extent, better understanding of functional and hormonal alterations produced in the neuroendocrine system in women who have lost ovarian function because of therapeutic ovariectomy or menopause.
